# Correlation of red cell distribution width-to-albumin ratio combined with SOFA score with 28-day all-cause mortality in patients with acute pancreatitis: A retrospective study based on the MIMIC-IV database

**DOI:** 10.1371/journal.pone.0357579

**Published:** 2026-09-01

**Authors:** Jianlei Chen, Hua Li, Hongfei Wang, Zhiqiang He, Jingzhao Yin, Songsong Yu, Tianyu Wu, Xiaopeng Guan, Bowen Zheng, Depei Kong, Hao Li

**Affiliations:** 1 Department of Hepatobiliary and Pancreatic Surgery, the Affiliated Hospital of Yanbian University (Yanbian Hospital), Yanji, Jilin, China; 2 Yanji Center for Disease Control and Prevention, Yanji, Jilin, China; 3 7th Department of Surgery, Puning People’s Hospital, Puning, Guangdong, China; 4 Department of Breast and Thyroid Surgery, Meihe Hospital the First Hospital of Jilin University (Meihekou Central Hospital), Changchun, Jilin, China; Pescara General Hospital, ITALY

## Abstract

**Objectives:**

The objective of this study is to evaluate the effectiveness of the red cell distribution width-to-albumin ratio (RAR) in conjunction with the Sequential Organ Failure Assessment (SOFA) score for predicting 28-day all-cause mortality in patients with acute pancreatitis (AP).

**Methods:**

In this study, we conducted a retrospective analysis of clinical data from 702 patients with AP sourced from the MIMIC-IV (v3.1) database. We evaluated the relationship between the RAR-modified SOFA score and 28-day all-cause mortality using both univariate and multivariate Cox regression analyses. Furthermore, the optimal cutoff value was determined using the Youden index, and the predictive performance of the combined indicator was evaluated through Receiver Operating Characteristic (ROC) curves and Kaplan-Meier (K-M) survival analysis. Finally, we examined the robustness of our findings across different populations through subgroup analyses.

**Results:**

A total of 702 patients with AP admitted to the Intensive Care Unit (ICU) were included in this study, and their 28-day all-cause mortality was analyzed. Both univariate and multivariate Cox regression analyses indicated that the RAR-modified SOFA score served as an independent risk factor for 28-day all-cause mortality in ICU patients with AP. ROC curve analysis demonstrated that the RAR-modified SOFA score exhibited superior predictive ability compared to other scoring systems, including RAR and SOFA alone. Additionally, K-M survival curve analysis revealed that the mortality rate was significantly higher in the high-value group (RAR-modified SOFA score ≥ 12.69) compared to the low-value group (RAR-modified SOFA score < 12.69).

**Conclusions:**

The RAR-modified SOFA score significantly enhances the predictive accuracy for 28-day all-cause mortality in patients with AP.

## 1. Introduction

Acute pancreatitis (AP) is associated with serious health consequences and potentially life-threatening effects. It is characterized by epigastric pain, abdominal distension, nausea, and vomiting [1]. As a prevalent acute abdominal condition complicated by a systemic inflammatory response and persistent organ failure, AP can lead to tissue and organ damage through mechanisms involving oxidative stress and the release of pancreatic enzymes [2]. Approximately 20% of patients with AP develop severe acute pancreatitis (SAP), with a mortality rate as high as 40% [3,4]. Early and accurate assessment of disease severity is therefore essential for optimizing treatment strategies and improving survival. The aim of this study was to evaluate the effectiveness of the red cell distribution width-to-albumin ratio (RAR) in conjunction with the Sequential Organ Failure Assessment (SOFA) score for predicting 28-day all-cause mortality in patients with AP. We hypothesized that combining RAR with SOFA would yield superior predictive performance compared to either indicator alone.

Red cell distribution width (RDW) is a hematological index reflecting the heterogeneity of red blood cell volume, which is strongly associated with oxidative stress, inflammatory responses, and nutritional status. Similarly, albumin is a critical nutritional marker that declines during malnutrition or inflammatory stimuli. The RAR has emerged as a promising composite biomarker that mitigates physiological fluctuations to better predict disease severity in acute conditions. Meanwhile, the SOFA score is extensively utilized to evaluate multiorgan dysfunction in critically ill patients, demonstrating high specificity in predicting mortality for severe acute pancreatitis. Given that RAR reflects systemic inflammation and nutritional status while SOFA captures multiorgan dysfunction.

In recent years, researchers have investigated the prognostic significance of composite biomarkers such as the neutrophil/lymphocyte ratio [5], platelet/lymphocyte ratio [6], lactate/albumin ratio [7], and C-reactive protein/albumin ratio [8]. They have sought to integrate traditional scoring systems to enhance the predictive validity and stability for AP. Previous studies have indicated that both the RAR and SOFA scoring systems possess substantial advantages in predicting outcomes in AP [9]. However, to date, no research has explored the combination of the RAR and SOFA scoring systems to assess the prognosis of AP patients. Our results are consistent with and extend previous studies on RAR in AP. Donmez et al. reported that RAR is a useful marker for identifying biliary pancreatitis [10], and Wang et al. demonstrated its predictive capability for SAP [11]. A recent study by Chen et al. showed a nonlinear relationship between RAR and long-term mortality in AP [12]. Regarding combination approaches, Ren et al. proposed that RAR combined with the Bedside Index for Severity in Acute Pancreatitis (BISAP) improves severity prediction [13], and Pan et al. developed a nomogram incorporating RAR for 30-day mortality prediction [14]. However, to our knowledge, no study has yet integrated RAR with the SOFA score, a widely used organ failure assessment tool to predict short-term mortality in AP. The SOFA score has shown high specificity in SAP mortality prediction [15], but its AUC in AP is modest. By combining RAR with SOFA, the present study provides a novel, easy-to-use prognostic indicator for clinical practice.

## 2. Methods

### 2.1 Database introduction

The data utilized in this study were retrieved from the MIMIC-IV (v3.1) database, a large, publicly available dataset maintained by the Laboratory of Computational Physiology at the Massachusetts Institute of Technology (https://physionet.org/content/mimiciv/3.1/). This database contains detailed information on patients admitted to the intensive care unit (ICU) at Beth Israel Deaconess Medical Center between 2008 and 2022 [16]. To access the database, the study’s first author, Jianlei Chen, successfully completed the Collaborative Institutional Training Initiative course and passed the required examinations, including “Conflict of Interest” and “Data or Sample Study Only” (ID: 67992428).

### 2.2 Population selection criteria

**Fig 1:**
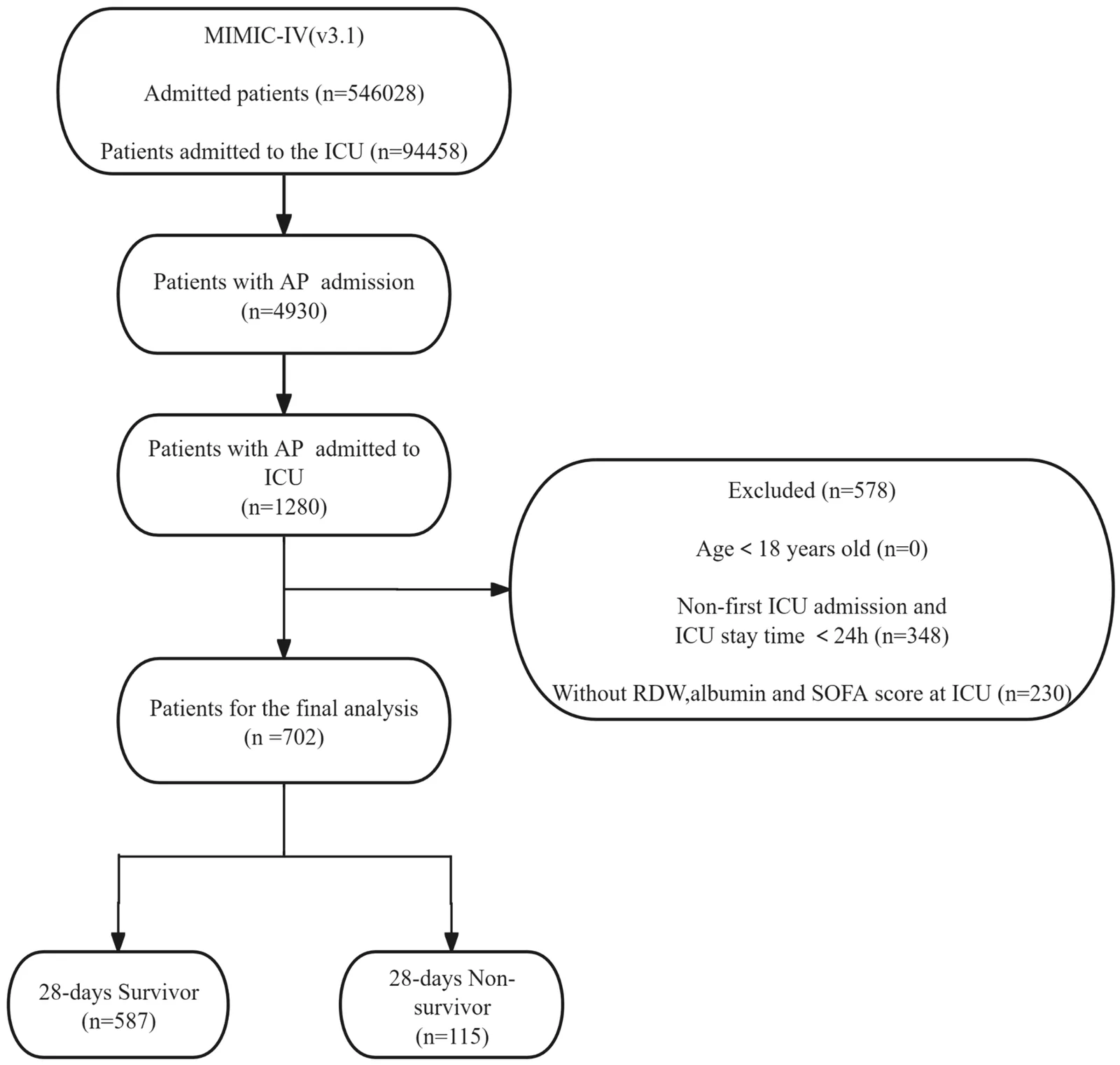
The flowchart details the inclusion and exclusion criteria and ultimately shows that data from 702 subjects were available for the final analysis.

The MIMIC-IV (v3.1) database recorded a total of 546028 admissions from 2008 to 2022, with 94458 of these patients admitted to the ICU. From this cohort, we identified 4930 patients with AP using the International Classification of Diseases, 9th Revision (ICD-9) code 577.0 and International Classification of Diseases, 10th Revision (ICD-10) codes K85-K85.92. Among these, 1280 patients with AP were admitted to the ICU. After a rigorous screening process, we excluded patients based on the following criteria: (1) individuals younger than 18 years at the time of their first admission; (2) patients with AP who were admitted to the ICU multiple times, retaining only data from their first admission; (3) those who remained in the ICU for less than 24 hours; and (4) patients admitted to the ICU without corresponding data on RDW, albumin levels, and SOFA scores. Consequently, the final number of patients included in this study was 702 (Fig 1).

### 2.3 Clinical and laboratory data extraction

The RAR-modified SOFA score, was selected as the primary study variable, with 28-day all-cause mortality as the primary outcome. Data extraction was conducted using PostgreSQL (version 17.3). To minimize the potential impact of subsequent treatments on RDW and albumin levels, baseline data were collected at the first test following admission to the ICU. The following data were systematically collected: demographic information, vital signs, clinical treatments, comorbidities, laboratory markers, and various scoring systems, including the SOFA score, the Glasgow Coma Scale (GCS), the Oxford Acute Severity of Illness Score (OASIS), and the Systemic Inflammatory Response Syndrome Score (SIRS). Comorbidities including acute kidney injury (AKI), congestive heart failure (CHF), atrial fibrillation (AF), diabetes, obesity, hypertension, sepsis were extracted from the MIMIC-IV database using ICD-9 and ICD-10 codes that represent the diagnosis made by the attending physician. We did not apply additional diagnostic criteria.

### 2.4 Statistical analysis

The primary outcome of the study was defined as all-cause mortality within 28 days of ICU admission. The patient cohort was divided into two groups: the 28-day ICU admission survivor group (n = 587) and the 28-day ICU admission non-survivor group (n = 115). Descriptive statistics were performed for all patients. Categorical variables were reported as proportions (%) and analyzed using the χ² test or Fisher’s exact test, as appropriate. Normally distributed continuous variables were expressed as mean ± standard deviation (SD) and compared using Student’s t-test or one-way ANOVA. For continuous variables that did not conform to a normal distribution, data were presented as median and interquartile range (IQR) and analyzed using the Kolmogorov-Smirnov test. Continuous variables with missing values of less than 2% (such as temperature, neutrophils, lymphocytes, alanine aminotransferase, aspartate aminotransferase, total bilirubin (TBIL), serum creatinine, serum calcium, international normalized ratio (INR), SOFA score, and GCS) had missing values replaced with the mean or median of the respective variable.

The association between the RAR-modified SOFA score and 28-day mortality was assessed through univariate Cox regression analysis to identify potential risk factors. Covariates and factors with a *p* value of less than 0.1 were included in a multivariate Cox regression analysis. Recognizing that confounding is a significant concern in multifactorial regression analyses, both unadjusted and multivariate-adjusted models were employed to ensure the stability and robustness of the results. ROC analyses were conducted to evaluate the predictive ability of the RAR-modified SOFA score, RAR alone, and various other scoring systems for predicting 28-day mortality at the time of ICU admission. This included assessments of sensitivity, specificity, and calculation of the AUC. Pairwise comparisons of AUCs between the RAR-modified SOFA score and other scoring systems were performed using DeLong’s test for two correlated ROC curves. The Youden Index was utilized to determine the optimal thresholds for the RAR-modified SOFA score, categorizing these scores into high and low value groups. Subsequently, unadjusted survival curves were plotted using the K-M method, and comparisons between the two groups were made using the log-rank test. Finally, subgroup and interaction analyses were performed to investigate the effects of the RAR-modified SOFA score across different subgroups.

All statistical analyses were performed using R software version 4.2.2 and Free Statistics software version 2.1.1. A two-tailed test was used and a *p* value of 0.05 or less was considered statistically significant.

### 2.5 Definition of the RAR-modified SOFA score

RAR-modified SOFA = SOFA score + RAR value. This is a simple arithmetic addition, not a regression-coefficient-weighted composite. By preserving the SOFA score in its entirety, clinicians can continue to interpret the organ-dysfunction component while appreciating the incremental information conveyed by RAR. The direct addition allows rapid bedside calculation without complex transformations or dedicated tools.

### 2.6 Definition of acute pancreatitis severity

We defined SAP as AP accompanied by organ failure and/or pancreatic necrosis, according to the 2012 revision of the Atlanta classification of acute pancreatitis [17]. Organ failure was assessed using the SOFA score, with a score of ≥ 3 in any system defined as organ failure [18]. From the 702 patients, we further identified those with acute pancreatitis complicated by organ failure (defined as a SOFA score ≥ 3 in any organ system) and those with pancreatic necrosis (ICD codes: K8501, K8502, K8511, K8512, K8521, K8522, K8531, K8532, K8581, K8582, K8591, K8592), yielding a final cohort of 239 patients with severe acute pancreatitis.

## 3. Results

### 3.1 Baseline characteristics of study participants

A total of 702 patients were enrolled in this study, with baseline demographic and clinical characteristics compared between the groups (Table 1). Among the participants, 587 (83.6%) were classified in the 28-day survival group, while 115 (16.4%) were in the non-survival group. Among them, 239 (34.0%) were classified as SAP and 463 (66.0%) as non-SAP. Notably, the prevalence of SAP was significantly higher in the 28-day non-survivor group compared with the survivor group (71.3% [82/115] vs 26.7% [157/587], *p* < 0.001). The mean age of the patients was 58.3 ± 17.7 years, with a gender distribution of 306 (43.6%) females and 396 (56.4%) males. The cohort predominantly consisted of white individuals, with 430 (61.3%) enrolled. The results indicated that the RAR-modified SOFA score was significantly higher in the non-survival group compared to the survival group (14.7 ± 4.8 vs. 10.2 ± 3.9, *p* < 0.001). Furthermore, patients in the 28-day non-survival group exhibited lower body temperature, heart rate (HR), systolic blood pressure (SBP), diastolic blood pressure (DBP), mean arterial pressure (MAP), and oxygen saturation (SpO2) compared to those in the survival group (*p* < 0.05). Significant abnormalities were observed in laboratory indices, including white blood cell (WBC), RDW, platelet count, hemoglobin levels, TBIL, serum creatinine, blood urea nitrogen (BUN), potassium, albumin, and international normalized ratio (*p* < 0.05). Among comorbidities, the prevalence of AKI, sepsis, and AF was significantly higher in the non-survival group (*p* < 0.05). Regarding therapeutic interventions, the proportion of patients receiving continuous renal replacement therapy (CRRT), vasopressor medications, and octreotide was notably higher in the non-surviving group (*p* < 0.001). Additionally, the special scoring systems indicated significantly higher scores for OASIS and SOFA in the non-surviving group, and significantly lower scores for GCS in the non-surviving group (*p* < 0.05). No significant differences were found in the remaining covariates between the two groups (*p* > 0.05).

**Table 1:** Baseline characteristics of participants.

Variables	ALL patients (n = 702)	28-d survivors (n = 587)	28-d non-survivors (n = 115)	*p*
Gender, n (%)				0.143
Female	306 (43.6)	263 (44.8)	43 (37.4)	
male	396 (56.4)	324 (55.2)	72 (62.6)	
Age (years)	58.3 ± 17.7	58.3 ± 17.5	58.8 ± 18.8	0.779
Race, n (%)				0.616
White	430 (61.3)	361 (61.5)	69 (60)	
Black	55 (7.8)	45 (7.7)	10 (8.7)	
Asian	26 (3.7)	24 (4.1)	2 (1.7)	
Other	191 (27.2)	157 (26.7)	34 (29.6)	
HR (beats/min)	102.4 ± 22.1	103.5 ± 22.3	96.7 ± 20.1	**0.003**
SBP (mmHg)	125.3 ± 26.3	127.2 ± 26.1	115.9 ± 25.5	**< 0.001**
DBP (mmHg)	72.3 ± 19.5	73.6 ± 19.3	66.1 ± 19.3	**< 0.001**
MAP (mmHg)	86.0 ± 19.7	87.0 ± 19.3	80.5 ± 21.1	**0.001**
RR (beats/min)	22.3 ± 6.9	22.2 ± 6.8	22.8 ± 7.4	0.402
SpO2 (%)	95.8 ± 4.5	96.0 ± 3.9	94.9 ± 6.9	**0.019**
Temperature (℃)	36.9 ± 1.0	37.0 ± 1.0	36.4 ± 1.1	**< 0.001**
Neutrophils (×10^9/L)	11.3 (9.0, 15.0)	11.3 (8.9, 14.5)	11.3 (9.5, 17.1)	0.117
Lymphocytes (×10^9/L)	1.1 ± 1.1	1.1 ± 0.7	1.3 ± 2.0	0.13
WBC (×10^9/L)	13.3 (9.1, 19.1)	12.9 (8.9, 18.6)	15.3 (9.8, 21.1)	**0.019**
RDW (%)	15.3 ± 2.4	14.9 ± 2.0	16.9 ± 3.3	**< 0.001**
RBC (×10^12/L)	3.7 ± 0.9	3.7 ± 0.8	3.5 ± 1.0	0.053
Platelet (×10^9/L)	180.5 (125.0, 264.8)	185.0 (130.5, 272.0)	152.0 (100.0, 244.0)	**< 0.001**
Hemoglobin (g/dL)	11.2 ± 2.5	11.3 ± 2.5	10.7 ± 2.7	**0.024**
ALT (IU/L)	235.3 ± 882.7	236.9 ± 931.0	227.2 ± 579.2	0.915
AST (IU/L)	444.4 ± 1790.5	392.0 ± 1591.6	711.8 ± 2569.9	0.08
TBIL (mg/dL)	3.6 ± 6.6	2.8 ± 5.0	7.5 ± 10.8	**< 0.001**
Glucose (mg/dL)	157.0 ± 112.0	157.3 ± 112.5	155.7 ± 109.5	0.888
Serum creatinine (mg/dL)	1.8 ± 1.8	1.6 ± 1.7	2.6 ± 2.2	**< 0.001**
BUN (mg/dL)	29.3 ± 25.5	25.9 ± 22.3	46.4 ± 32.6	**< 0.001**
Calcium (mg/dL)	7.8 ± 1.2	7.8 ± 1.2	7.9 ± 1.2	0.662
Potassium (mmol/L)	4.2 ± 0.8	4.1 ± 0.8	4.3 ± 0.8	**0.037**
Sodium (mmol/L)	138.0 ± 6.2	137.9 ± 5.8	138.9 ± 7.7	0.096
Albumin (g/dl)	2.8 ± 0.6	2.9 ± 0.6	2.7 ± 0.7	**0.001**
INR	1.3 (1.2, 1.6)	1.3 (1.2, 1.5)	1.6 (1.3, 2.1)	**< 0.001**
AKI, n (%)				**< 0.001**
NO	142 (20.2)	138 (23.5)	4 (3.5)	
YES	560 (79.8)	449 (76.5)	111 (96.5)	
Obesity, n (%)				0.148
NO	605 (86.2)	501 (85.3)	104 (90.4)	
YES	97 (13.8)	86 (14.7)	11 (9.6)	
Sepsis, n (%)				**< 0.001**
NO	181 (25.8)	167 (28.4)	14 (12.2)	
YES	521 (74.2)	420 (71.6)	101 (87.8)	
CHF, n (%)				0.17
NO	586 (83.5)	495 (84.3)	91 (79.1)	
YES	116 (16.5)	92 (15.7)	24 (20.9)	
Diabetes, n (%)				0.251
NO	500 (71.2)	413 (70.4)	87 (75.7)	
YES	202 (28.8)	174 (29.6)	28 (24.3)	
Hypertension, n (%)				0.634
NO	273 (38.9)	226 (38.5)	47 (40.9)	
YES	429 (61.1)	361 (61.5)	68 (59.1)	
AF, n (%)				**0.005**
NO	551 (78.5)	472 (80.4)	79 (68.7)	
YES	151 (21.5)	115 (19.6)	36 (31.3)	
ERCP, n (%)				0.351
NO	682 (97.2)	572 (97.4)	110 (95.7)	
YES	20 (2.8)	15 (2.6)	5 (4.3)	
Ventilator, n (%)				0.148
NO	97 (13.8)	86 (14.7)	11 (9.6)	
YES	605 (86.2)	501 (85.3)	104 (90.4)	
CRRT, n (%)				**< 0.001**
NO	570 (81.2)	509 (86.7)	61 (53)	
YES	132 (18.8)	78 (13.3)	54 (47)	
Vasopressor, n (%)				**< 0.001**
NO	568 (80.9)	508 (86.5)	60 (52.2)	
YES	134 (19.1)	79 (13.5)	55 (47.8)	
Octreotide, n (%)				**< 0.001**
NO	649 (92.5)	554 (94.4)	95 (82.6)	
YES	53 (7.5)	33 (5.6)	20 (17.4)	
Omeprazole, n (%)				0.657
NO	566 (80.6)	475 (80.9)	91 (79.1)	
YES	136 (19.4)	112 (19.1)	24 (20.9)	
Metoprolol, n (%)				0.773
NO	468 (66.7)	390 (66.4)	78 (67.8)	
YES	234 (33.3)	197 (33.6)	37 (32.2)	
GCS	14.5 ± 1.1	14.6 ± 1.1	14.2 ± 1.4	**0.003**
OASIS	35.3 ± 9.3	34.2 ± 9.0	40.7 ± 9.2	**< 0.001**
SIRS	3.1 ± 0.8	3.1 ± 0.8	3.2 ± 0.8	0.12
SOFA	5.2 ± 3.5	4.7 ± 3.3	7.8 ± 3.7	**< 0.001**
SAP, n (%)				**< 0.001**
NO	463 (66.0)	430 (73.3)	33 (28.7)	
YES	239 (34.0)	157 (26.7)	82 (71.23)	
RAR	5.7 ± 1.8	5.5 ± 1.6	6.9 ± 2.4	**< 0.001**
RAR-modified SOFA	10.9 ± 4.4	10.2 ± 3.9	14.7 ± 4.8	**< 0.001**

Abbreviations: HR, Heart Rate; SBP, Systolic Blood Pressure; DBP, Diastolic Blood Pressure; MAP, Mean Arterial Pressure; RR, Respiratory Rate; Oxygen Saturation, SpO2; WBC; White Blood Cell; RDW, Red Blood Cell Distribution Width; RBC, Red Blood Cell; ALT, Alanine Aminotransferase; AST, Aspartate Aminotransferase; TBIL, Total Bilirubin; BUN, Blood Urea Nitrogen; INR, International Normalized Ratio; AKI, Acute Kidney Injury; CHF, Congestive Heart Failure; AF, Atrial Fibrillation; ERCP, Endoscopic Retrograde Cholangiopancreatography; CRRT, Continuous Renal Replacement Therapy; GCS, Glasgow Coma Score; OASIS, Oxford Acute Severity of Illness Score; SIRS, Systemic Inflammatory Response Syndrome; SOFA, Sequential Organ Failure Assessment; RAR, Red Blood Cell Distribution Width/Albumin Ratio.

### 3.2 Association of RAR-modified SOFA score with all-cause mortality 28 days after ICU admission

**Fig 2:**
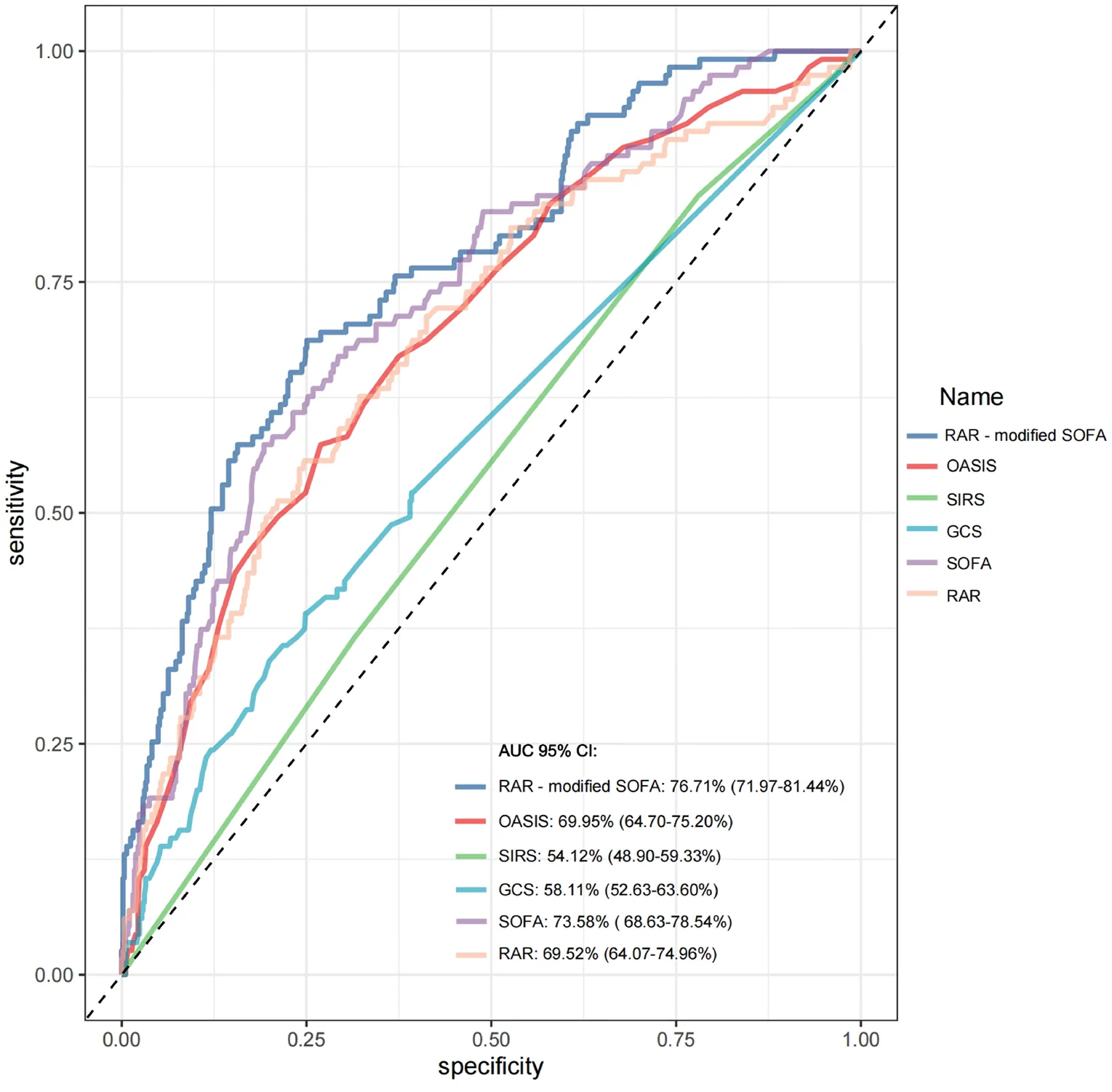
ROC analysis of RAR-modified SOFA for predicting 28-day mortality in acute pancreatitis. OASIS, Oxford Acute Severity of Illness Score; SIRS, Systemic Inflammatory Response Syndrome; GCS, Glasgow Coma Score; SOFA, Sequential Organ Failure Assessment; RAR, Red Blood Cell Distribution Width/Albumin Ratio.

**Fig 3:**
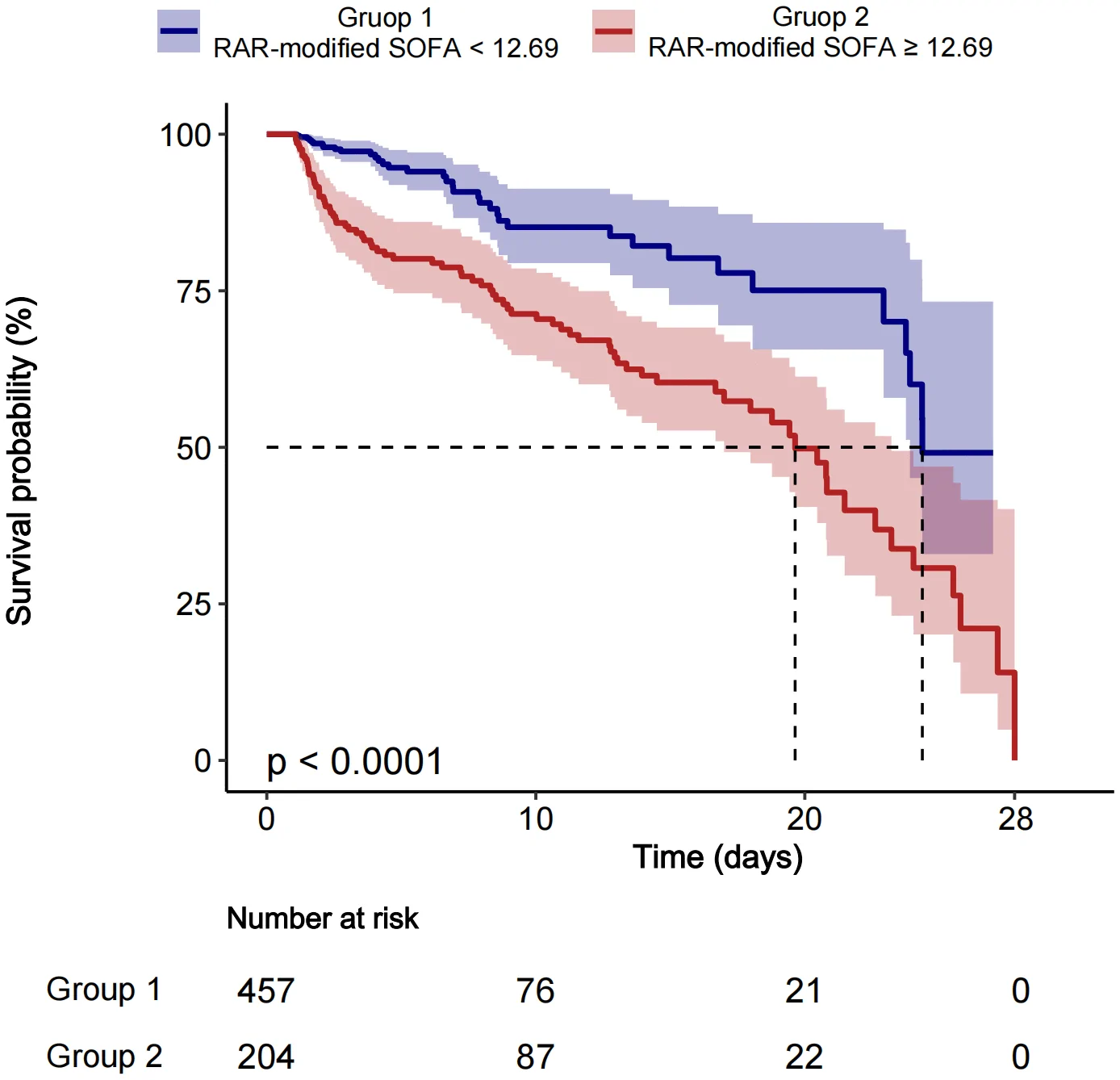
K-M survival analysis curves for all-cause mortality within 28-day of Intensive Care Unit admission. Group 1, RAR-modified SOFA < 12.69; Group 2, RAR-modified SOFA ≥ 12.69. The table below the curves presents the number of patients at risk at each time point (0, 10, 20 and 28 days).

Covariates with significant differences (*p* < 0.05) in baseline characteristics were included in the univariate Cox regression analysis. This analysis revealed that the unadjusted RAR-modified SOFA score was significantly associated with all-cause mortality within 28 days of ICU admission (HR, 1.13; 95% CI, 1.08–1.17; *p* < 0.001). Univariate Cox regression analysis (Table 2) revealed that, in addition to the RAR-modified SOFA score, multiple factors were significantly associated with 28-day all-cause mortality. These included vital signs (HR, SBP, DBP, MAP, and temperature), laboratory parameters (RDW, platelet, hemoglobin, TBIL, serum creatinine, BUN, and INR), therapeutic interventions (CRRT, vasopressor, and octreotide), as well as other scoring systems (OASIS, SOFA, and RAR). After accounting for potential confounding and avoiding multicollinearity, the multivariate model was adjusted for gender, age, race, diabetes, CHF, and obesity. In this adjusted model, the RAR-modified SOFA score remained an independent risk factor for 28-day mortality (HR, 1.13; 95% CI, 1.08–1.17; *p* < 0.001) (Table 3). Additionally, we plotted ROC curves for RAR-modified SOFA score, RAR alone, and four different scoring systems to predict all-cause mortality in patients with AP within 28 days of ICU admission **(**Fig 2 and S1-S5 Fig), with the corresponding ROC curve information presented in (Table 4). The results indicated that the AUC for RAR-modified SOFA was 76.71% (95% CI: 71.97–81.44%). DeLong’s test for correlated ROC curves showed that this AUC was significantly larger than that of RAR alone, which had an AUC of 69.52% (95% CI: 64.07–74.96%, *p* = 0.004). Similarly, the RAR-modified SOFA also demonstrated a significantly superior AUC compared to the SOFA score alone, which achieved an AUC of 73.58% (95% CI: 68.63–78.54%, *p* = 0.008). Furthermore, the predictive performance of RAR-modified SOFA was not inferior to that of other scoring systems, including OASIS (AUC: 69.95%, *p* = 0.027 for comparison RAR-modified SOFA), SIRS (AUC: 54.12%, *p* < 0.001), and GCS (AUC: 58.11%, *p* < 0.001). Subsequently, we determined an optimal cutoff value of 12.69 for the RAR-modified SOFA score, which yielded a sensitivity of 68.7% and a specificity of 74.96%. Based on this cutoff value, patients with AP were categorized into a low-value group (RAR-modified SOFA < 12.69, n = 476) and a high-value group (RAR-modified SOFA ≥ 12.69, n = 226). K-M survival analysis curves were also plotted (Fig 3), demonstrating that the mortality rate in the high-value group was significantly higher than that in the low-value group (*p* < 0.0001).

**Table 2:** Univariate Cox analysis of risk factors for death within 28-d in patients.

Variables	Univariate COX	
	HR (95% CI)	*p*
HR (beats/min)	0.99 (0.98,0.99)	**< 0.001** **< 0.001** **0.001** **0.004** 0.47 **< 0.001** 0.116 **< 0.001** **0.02** **0.008** **< 0.001** **< 0.001** **< 0.001** 0.095 0.304 **0.026** 0.139 0.597 0.073 **< 0.001** **< 0.001** **< 0.001** 0.168 **0.001** **< 0.001** **< 0.001** **< 0.001**
SBP (mmHg)	0.99 (0.98,0.99)	
DBP (mmHg)	0.98 (0.97,0.99)	
MAP (mmHg)	0.99 (0.98,1.00)	
SpO2 (%)	0.99 (0.96,1.02)	
Temperature (℃)	0.66 (0.58,0.76)	
WBC (×10^9/L)	1.02 (1.00,1.03)	
RDW (%)	1.18 (1.12,1.24)	
Platelet (×10^9/L)	1.00 (0.99,1.00)	
Hemoglobin (g/dL)	0.90 (0.84,0.97)	
TBIL (mg/dL)	1.04 (1.03,1.06)	
Serum creatinine (mg/dL)	1.20 (1.12,1.30)	
BUN (mg/dL)	1.02 (1.01,1.02)	
Potassium (mmol/L)	1.18 (0.97,1.44)	
Albumin (g/dl)	0.86 (0.65,1.14)	
INR	1.13 (1.01,1.27)	
AKI, n (%)	2.16 (0.78,6.02)	
Sepsis, n (%)	0.85 (0.47,1.54)	
AF, n (%)	1.44 (0.97,2.13)	
CRRT, n (%)	2.17 (1.49,3.17)	
Vasopressor, n (%)	2.10 (1.44,3.07)	
Octreotide, n (%)	2.39 (1.47,3.88)	
GCS	0.93 (0.84,1.03)	
OASIS	1.03 (1.01,1.05)	
SOFA	1.13 (1.08,1.18)	
RAR	1.17 (1.09,1.25)	
RAR-modified SOFA	1.13 (1.08,1.17)	

Abbreviations: HR (95% CI), Hazard Ratio (95% Confidence Interval); HR, Heart Rate; SBP, Systolic Blood Pressure; DBP, Diastolic Blood Pressure; MAP, Mean Arterial Pressure; Oxygen Saturation, SpO2; WBC; White Blood Cell; RDW, Red Blood Cell Distribution Width; TBIL, Total Bilirubin; BUN, Blood Urea Nitrogen; INR, International Normalized Ratio; AKI, Acute Kidney Injury; AF, Atrial Fibrillation; CRRT, Continuous Renal Replacement Therapy; GCS, Glasgow Coma Score; OASIS, Oxford Acute Severity of Illness Score; SOFA, Sequential Organ Failure Assessment; RAR, Red Blood Cell Distribution Width/Albumin Ratio.

**Table 3:** Multivariate Cox analysis of risk factors for death in patients within 28-d.

Variable	28-day mortality		
	HR	95% CI	*p*
Crude Model	1.13	1.08 ~ 1.17	**<0.001**
Adjusted Model	1.13	1.08 ~ 1.17	**<0.001**

Abbreviations: HR, Hazard Ratio; CI, Confidence Interval.

Crude Model was unadjusted.

Adjusted Model was adjusted for gender, age, race, diabetes, CHF, and obesity.

**Table 4:** Information of ROC curves in Fig 2.

Variables	AUC	95% CI	Threshold	Sensitivity	Specificity
RAR-modified SOFA	76.71%	71.97% ~ 81.44%	12.69	0.69	0.75
OASIS	69.95%	64.70% ~ 75.20%	39.50	0.57	0.73
SIRS	54.12%	48.90% ~ 59.33%	2.50	0.84	0.22
GCS	58.11%	52.63% ~ 63.60%	14.63	0.39	0.75
SOFA	73.58%	68.63% ~ 78.54%	7.57	0.57	0.81
RAR	69.52%	64.07% ~ 74.96%	6.20	0.56	0.75

Abbreviations: AUC, area under the curve; CI, Confidence Interval; OASIS, Oxford Acute Severity of Illness Score; SIRS, Systemic Inflammatory Response Syndrome; GCS, Glasgow Coma Score; SOFA, Sequential Organ Failure Assessment; RAR, Red Blood Cell Distribution Width/Albumin Ratio.

### 3.3 Subgroup analysis

**Fig 4:**
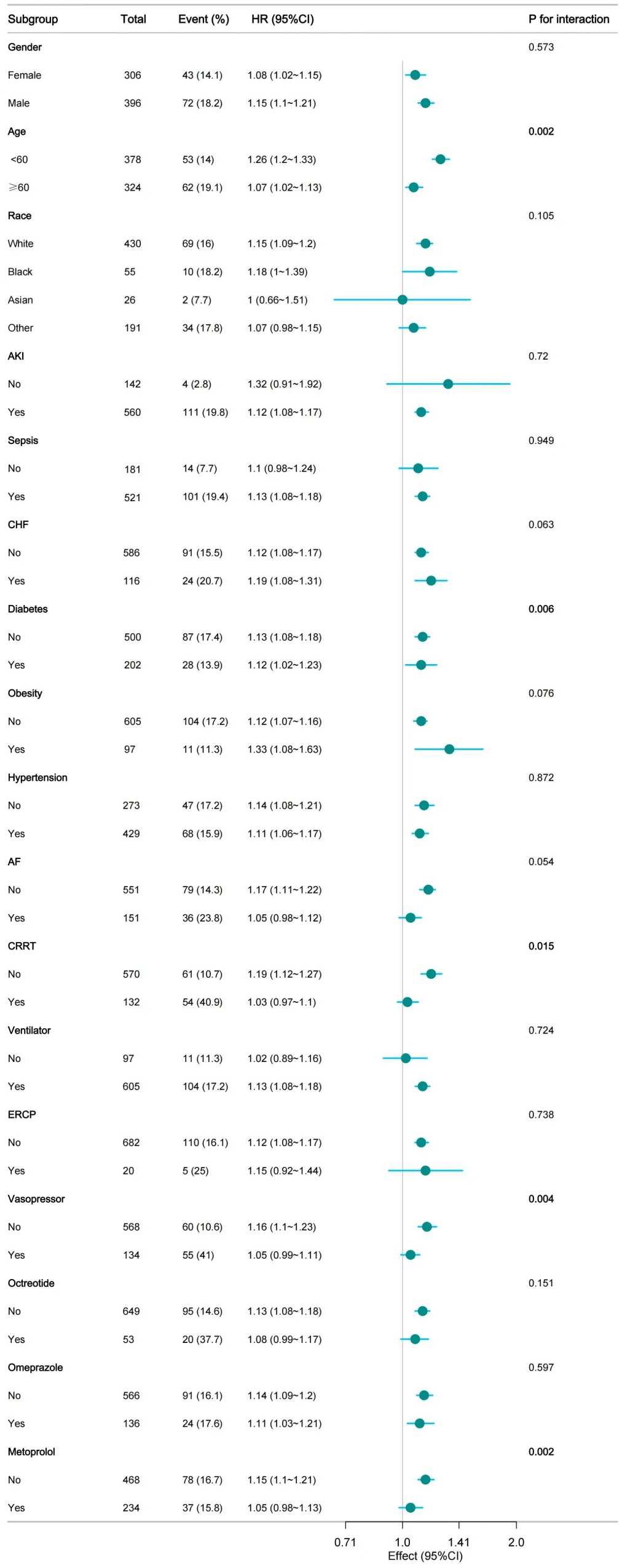
Forest plot for subgroup analysis of the relationship between Intensive Care Unit mortality and RAR-modified SOFA. AKI, Acute Kidney Injury; CHF, Congestive Heart Failure; AF, Atrial Fibrillation; CRRT, Continuous Renal Replacement Therapy; ERCP, Endoscopic Retrograde Cholangiopancreatography; RAR, Red cell distribution width/Albumin Ratio; SOFA, Sequential Organ Failure Assessment. *p* values less than 0.05 is shown in bold.

To assess the relationship between the RAR-modified SOFA score and 28-day all-cause mortality in patients with AP, we conducted subgroup and interaction analyses considering potential confounders such as gender, age, race, comorbidities, and clinical treatments (Fig 4). The findings indicated that the effect size of RAR-modified SOFA score on 28-day all-cause mortality in AP patients remained robust and reliable, with no significant interactions observed between RAR and most subgroups (*p* > 0.05). However, we did identify some interactions among participants related to age, diabetes, CRRT, vasopressor medications, and metoprolol (*p* < 0.05). Nonetheless, due to the multiple testing conducted and the consistent direction of the associations, these results may not be clinically significant.

### 3.4 Sensitivity analysis

We conducted a sensitivity analysis in 239 patients with SAP. Multivariate Cox regression analysis demonstrated that the RAR-modified SOFA score remained an independent prognostic risk factor, with significant associations observed across all models: the crude model (HR, 1.10; 95% CI, 1.05–1.16; *p* < 0.001) and the adjusted model (HR, 1.09; 95% CI, 1.04–1.15; *p* = 0.001). In addition, the K-M survival curve results are also consistent with our previous findings. (S1 Table and S6 Fig)

## 4. Discussion

In this retrospective study, we analyzed clinical data from 702 patients with AP sourced from the MIMIC-IV (v3.1) database and integrated the traditional SOFA scoring system with the RAR metric. Our findings indicated that the RAR-modified SOFA score was an independent risk factor for all-cause mortality within 28 days of ICU admission, exhibiting an AUC value of 76.71%. This performance was significantly superior to that of the RAR (69.52%) and SOFA score (73.58%) when used individually. Furthermore, the RAR-modified SOFA score achieved a higher AUC (76.71%) compared to other conventional scoring systems including OASIS (69.95%), SIRS (54.12%), and GCS (58.11%), indicating its improved predictive performance for 28-day mortality. Additionally, K-M survival analysis revealed that the 28-day all-cause mortality rate for patients with a RAR-modified SOFA score ≥ 12.69 was significantly higher than that for those with a score < 12.69. Our conclusions were further substantiated by subgroup analyses and interaction assessments.

RDW is a hematological index that reflects the heterogeneity of red blood cell volume. Traditionally, RDW has been utilized as one of the diagnostic indices for anemia [19]. However, in recent years, researchers have identified that RDW also holds significant clinical utility in various acute and chronic conditions, including cardiovascular disease [20], chronic obstructive pulmonary disease [21], sepsis [22], and chronic liver disease [23]. A retrospective study has demonstrated that RDW is particularly advantageous for predicting SAP. This phenomenon may be attributed to the strong association between elevated RDW and factors such as oxidative stress, inflammatory responses, and the nutritional status of tissue cells [19,24]. The process of autodigestion in AP can lead to damage of the pancreatic follicles, which in turn stimulates the release of inflammatory cells, predominantly neutrophils and macrophages, within the pancreatic parenchyma. Furthermore, the activation of oxidative stress in pancreatic alveolar cells can exacerbate inflammatory responses during AP [25]. Albumin, a common nutritional marker in blood tests, serves as an indicator of malnutrition or inflammatory stimuli when its levels decline [26]. Additionally, albumin has been shown to mitigate inflammatory responses by promoting the synthesis of lipoproteins and hemolysins [27]. Patients with AP utilize significant amounts of albumin in their efforts to enhance the body’s defense mechanisms against disease. However, the complex pathophysiological environment can render both RDW and albumin susceptible to its effects, thus diminishing their predictive capacity for disease severity. Consequently, researchers have begun to investigate the utility of the RAR in the diagnosis and management of AP. By examining the fluctuation of this ratio, they aim to mitigate the influence of the physiological mechanisms underlying the disease on prognostic predictions. A recent retrospective study involving 499 patients with AP highlighted the significance of RAR not only in predicting short-term mortality but also in long-term mortality among these patients [12]. The predictive capacity of RAR for short-term mortality, as determined by ROC curves, was found to have an AUC of 69.52%, which is comparable to the findings of the aforementioned study (AUC: 70.3%), thereby reinforcing the reliability of our results. Importantly, this study also integrated the BISAP score with RAR, achieving an AUC of 76.4% for predicting 28-day all-cause mortality, thus enhancing the predictive ability compared to the BISAP score alone (AUC: 73.5%). Prior to this retrospective study, a letter to the editor by Ren et al. noted that the combination of RAR and the BISAP score demonstrates considerable clinical utility in predicting the severity of AP [13].

The SOFA score is a scoring system designed to evaluate the functional status of organs in critically ill patients. Since its introduction in the 1990s, it has been extensively utilized across a range of critical conditions for daily monitoring, including sepsis [28], acute kidney failure [29], acute heart failure [30], and multiple organ dysfunction syndrome (MODS). In cases of AP, early untreated or concomitant infectious pancreatic necrosis frequently progresses to SAP. The pathophysiological mechanisms underlying systemic injury associated with SAP remain inadequately understood. Current research suggests that several key mediators involved in AP significantly contribute to organ failure. For instance, the synthesis of inflammatory mediators facilitates the infiltration of neutrophils into the pancreas. Additionally, an increase in lysosomes, zymogen granules, and oxidized glutathione can enhance the production of trypsin, which may damage acinar cells. Furthermore, the release of pancreatic lipase can lead to the destruction of adipose tissue, thereby exacerbating localized necrosis of the pancreatic parenchyma [2]. The application of the SOFA scoring system for the timely evaluation of multiorgan injury severity caused by AP has emerged as a critical factor in guiding treatment strategies. A retrospective study conducted by Teng et al. demonstrated that the SOFA score exhibits high specificity in predicting mortality in SAP, with an AUC value of 98.9% [15]. This finding underscores the relevance of the SOFA score as a quantitative evaluation tool aligned with the pathogenesis of SAP, as reported by Buter et al [31]. In another investigation involving 55 pediatric patients with AP, the Pediatric SOFA, specifically modified for children, proved to be highly significant in assessing the severity of AP-related multiorgan damage [32]. In our study, we also evaluated the predictive power of the SOFA score; although the AUC value of 73.58% differed from the findings reported by Teng et al., it still demonstrated a reasonably strong predictive capability for short-term mortality in AP cases. Early dynamic assessment of AP-induced SIRS and multiorgan dysfunction using the SOFA scoring system is critical for guiding a comprehensive, multi-pronged treatment strategy. First, timely identification of patients at risk of developing SAP facilitates early triage to a specialized center or ICU, which is associated with reduced mortality. Goal-directed fluid resuscitation, early enteral nutrition, and organ support therapy are implemented to attenuate organ injury, improve outcomes, and prevent clinical deterioration in patients with SAP [33–35].

Our study builds upon previous literature and presents an exploratory proposal to integrate the RAR with the SOFA score as a means to synthesize assessments of inflammatory response, oxidative stress, and nutritional status monitoring [36]. Prior studies have established a relationship between the BISAP modified by RAR and AP. Furthermore, Pan et al. investigated the feasibility of enhancing short-term mortality prediction in AP by developing a novel RAR-based column chart that incorporated several high-risk parameters associated with the condition [14]. To date, no study has integrated the RAR with the SOFA score to predict short-term mortality in AP. Although the SOFA score is widely used for organ failure assessment and RAR has shown prognostic value in AP, their combined application has not been explored. Therefore, this study evaluated the predictive performance of the RAR-modified SOFA score for 28-day all-cause mortality in patients with AP, thereby filling this research gap.

Classification of SAP based on the Atlanta criteria itself carries prognostic value, but the RAR-modified SOFA score offers several complementary advantages. First, SAP classification is a categorical variable, whereas the RAR-modified SOFA score provides a continuous risk gradient, enabling more granular risk stratification. This mechanistic complementarity is supported by our sensitivity analysis restricted to SAP patients (n = 239). Second, from a clinical workflow perspective, the diagnosis of SAP may require imaging confirmation of pancreatic necrosis or formal documentation of persistent organ failure, which can entail delays. In contrast, the RAR-modified SOFA score can be calculated immediately upon ICU admission using routinely available laboratory and clinical data, facilitating early risk assessment.

Although the SOFA score and RAR originate from different measurement scales, with SOFA being an ordinal organ dysfunction score and RAR a continuous laboratory ratio, their combination through simple addition worth exploring. We contend that this approach does not fundamentally compromise the clinical interpretability of SOFA for three reasons: First, the SOFA component remains intact and directly interpretable within the composite, allowing clinicians to readily assess the degree of organ dysfunction. Second, despite its different scale, RAR contributes incremental prognostic information that reflects systemic inflammation and nutritional status, dimensions that SOFA alone cannot capture, mirroring the common clinical practice of integrating laboratory values with scoring systems. Third, the combined score offers superior discriminative performance while maintaining practical utility, as both components are routinely available in the ICU setting.

Our study has several limitations that should be acknowledged. First, all data were derived from the MIMIC-IV (v3.1) database, which represents a single-center retrospective cohort. Consequently, we were unable to establish a detailed causal relationship between the RAR and the SOFA scoring system. Second, our analysis was limited to laboratory indicators collected at the time of the patients’ initial admission to the ICU, without accounting for potential dynamic changes over time. Future studies should aim to evaluate RAR at multiple time points to further validate the clinical applicability of combining RAR with SOFA scores. Lastly, the patient data included in our study span the period from 2008 to 2022. Given advancements in medical care and innovations in therapeutic approaches during this time frame, there is no guarantee that treatment regimens were consistently applied across all patients. This variability may have influenced the study outcomes, but we were unable to assess its impact. These limitations highlight the need for further investigation to strengthen the robustness of our findings and support their translation into clinical practice.

## 5. Conclusions

Our study found that the RAR-modified SOFA score is an independent risk factor for 28-day all-cause mortality. The combination significantly improved predictive performance compared to RAR or SOFA alone. These findings are clinically relevant because RAR reflects oxidative stress, inflammation, and nutritional status, while SOFA captures multiorgan dysfunction, both key pathophysiological drivers of mortality in SAP. By integrating these two dimensions, the RAR-modified SOFA score provides a more comprehensive risk assessment tool that can be easily calculated from routine laboratory and clinical data at ICU admission.

## Supporting information

S1 Fig ROC analysis was used to compare the predictive performance of RAR-modified SOFA versus OASIS scoring systems for 28-day mortality in patients with AP. RAR, Red cell distribution width/Albumin Ratio; SOFA, Sequential Organ Failure Assessment; OASIS, Oxford Acute Severity of Illness Score; AP, Acute pancreatitis; The blue line represents RAR-modified SOFA; The red line represents OASIS.
(TIF)

S2 Fig ROC analysis was used to compare the predictive performance of RAR-modified SOFA versus SIRS scoring systems for 28-day mortality in patients with AP. RAR, Red cell distribution width/Albumin Ratio; SOFA, Sequential Organ Failure Assessment; SIRS, Systemic Inflammatory Response Syndrome; AP, Acute pancreatitis; The blue line represents RAR-modified SOFA; The red line represents SIRS.
(TIF)

S3 Fig ROC analysis was used to compare the predictive performance of RAR-modified SOFA versus GCS scoring systems for 28-day mortality in patients with AP. RAR, Red cell distribution width/Albumin Ratio; SOFA, Sequential Organ Failure Assessment; GCS, Glasgow Coma Scale; AP, Acute pancreatitis; The blue line represents RAR-modified SOFA; The red line represents GCS.
(TIF)

S4 Fig ROC analysis was used to compare the predictive performance of RAR-modified SOFA versus SOFA scoring systems for 28-day mortality in patients with AP. RAR, Red cell distribution width/Albumin Ratio; SOFA, Sequential Organ Failure Assessment; AP, Acute pancreatitis; The blue line represents RAR-modified SOFA; The red line represents SOFA.
(TIF)

S5 Fig ROC analysis was used to compare the predictive performance of RAR-modified SOFA versus RAR for 28-day mortality in patients with AP. RAR, Red cell distribution width/Albumin Ratio; SOFA, Sequential Organ Failure Assessment; The blue line represents RAR-modified SOFA; The red line represents RAR.
(TIF)

S6 Fig K-M survival analysis of 28-day all-cause mortality in patients with severe acute pancreatitis. Group 1, RAR-modified SOFA < 12.69; Group 2, RAR-modified SOFA ≥ 12.69. The table below the curves presents the number of patients at risk at each time point (0, 7, 14, 21 and 28 days).
(TIF)

S1 Table Multivariate Cox analysis of risk factors for 28-day mortality in patients with severe acute pancreatitis. HR, Hazard Ratio; CI, Confidence Interval. Crude Model was unadjusted; Adjusted Model was adjusted for gender, age, race, diabetes, CHF, and obesity.
(XLSX)
